# Significant variants of type 2 diabetes in the Arabian Region through an Integration of exome databases

**DOI:** 10.1371/journal.pone.0249226

**Published:** 2021-04-13

**Authors:** Kosuke Goto, Katsuhiko Mineta, Satoru Miyazaki, Takashi Gojobori

**Affiliations:** 1 King Abdullah University of Science and Technology (KAUST), Computational Bioscience Research Center (CBRC), Thuwal, Kingdom of Saudi Arabia; 2 Faculty of Pharmaceutical Sciences, Department of Medicinal and Life Sciences, Tokyo University of Science, Noda, Chiba, Japan; Universita degli Studi di Roma Tor Vergata, ITALY

## Abstract

Type 2 diabetes (T2D) is a major global health issue, and it has also become one of the major diseases in Arab countries. In addition to the exome databases that have already been established, whole exome sequencing data for the Greater Middle East are now available. To elucidate the genetic features of T2D in the Arabian Peninsula, we integrated two exome databases (gnomAD exome and the Greater Middle East Variome Project) with clinical information from the ClinVar. After the integration, we obtained 18 single nucleotide polymorphisms and found two statistically and clinically significant variants in two genes, *SLC30A8* rs13266634 and *KCNJ11* rs5219. Interestingly, the two genes are linked to the uptake of the metals, Zn and K respectively, which indicating the regional features of the genetic variants. The frequency of the risk allele of rs13266634 among individuals in the Arabian Peninsula was higher than among individuals in other regions. On the other hand, the frequency of the risk allele of rs5219 in the Arabian Peninsula was lower than that in other regions. We identified and characterized T2D-related variants that show unique tendencies in the Arabian Peninsula. Our analyses contribute to and provide guidance for the clinical research of T2D in the Arabian Peninsula.

## Introduction

Type 2 diabetes (T2D) is a disease characterized by insulin resistance. The risk factors of T2D include not only environmental factors but also genetic factors, such as a family history of diabetes and ethnicity [1]. Some genes, including *ABCC8* (ATP binding cassette subfamily C member 8), *CAPN10* (calpain 10), *SLC2A2* (solute carrier family 2 member 2), and *GCGR* (glucagon receptor) have been reported to be genetic factors associated with T2D [2]. T2D ranks among the major health issues worldwide, and it has also become one of the major diseases in Arab countries, mostly in high-income countries such as the Kingdom of Saudi Arabia and the United Arab Emirates [3]. The International Diabetes Federation estimates that the prevalence of diabetes in regions of the Middle East and North Africa region will increase from 12.8% (2019) to 15.7% (2045), and the expense for treating diabetes will increase from 24.9 billion USD to 38.6 billion USD [4].

With advancements in the development of sequencing technologies, many human genome projects for several populations are ongoing. These include the International HapMap Project [5], The Human Genome Diversity Project (HGDP) [6], and The 1000 Genomes project [7]. The Genome Aggregation Database (gnomAD) [8] is a resource of both genome and exome sequence data and gnomAD version 2.1.1 exome database provides genetic variants on eight populations (African/African-American, Ashkenazi Jewish, Finnish, Non-Finnish European, South Asian, East Asian, Latino/Admixed American, and Other). Although the populations are wide-ranging, none of the projects have provided much information on Middle Eastern populations.

In 2016, the Greater Middle Eastern Variome Project (GME Variome) was published [9]. The project provides genotype frequencies for seven populations in the greater Middle Eastern regions (Northeast Africa, Northwest Africa, the Arabian Peninsula, Israel, the Syrian Desert, the Turkish Peninsula, and Central Asia). When data from GME Variome are combined data from the gnomAD exome database, exome data corresponding to most of the world become available.

Several genome-wide association studies have been conducted on T2D [10–15], which have identified the single nucleotide polymorphisms (SNPs) associated with T2D. The information is stored in publicly available databases, such as the dbSNP and ClinVar. ClinVar provides not only SNP information but also clinical significance. The allele frequencies of some SNPs related to diseases (including T2D) are different among populations [16]. Unfortunately, most of the studies have been performed on the European and Asian populations, whereas studies on the Middle Eastern populations are limited [17–19].

To reveal the genotype features of individuals who live in the Middle Eastern regions, particularly the Arabian Peninsula, we integrated these two exome databases with the ClinVar database. We performed statistical analyses by comparing allele frequencies between the Arabian Peninsula and other regions.

## Materials and methods

### Integration of exome databases

The gnomAD version 2.1.1 exome data are available at https://gnomad.broadinstitute.org/downloads; and we downloaded data in the VCF format. The data included 17,209,972 variants from 125,748 samples of eight populations. The VCF file contained the rs-number, AN_POP for Allele Count, and AC_POP for Alt Allele Count for a population POP, where POP is one of the following: afr (African/African American), amr (Latino/Admixed American), asj (Ashkenazi Jewish), eas (East Asian), fin (Finnish), nfe (Non-Finish European), sas (South Asian), and oth (Other). We calculated the number of reference alleles as AN_POP–AC_POP for each variant per population.

Additionally, we downloaded GME Variome autosomal data from http://igm.ucsd.edu/gme/download.shtml. This project provides genotype frequencies of 669,953 variants on the autosomal chromosome for seven regions in the Greater Middle East (Northeast Africa, Northwest Africa, Arabian Peninsula, Israel, Syrian Desert, Turkish Peninsula, and Central Asia). The Arabian Peninsula included the following regions: Kuwait, Oman, Qatar, Kingdom of Saudi Arabia, the United Arab Emirates, and Yemen [9].

Since both databases are based on the human reference genome GRCh37/hg19, we merged the data when the chromosome number, nucleotide position, reference allele, and alternative allele were the same in both databases for each SNP. We included the alleles if the allele frequencies in each population were >0.01 to exclude rare variants. Because gnomAD data are based on populations, and GME Variome are based on their definitions; thus, we use “populations/regions” as a unit for this analysis. We disregarded the differences in allele frequencies among other populations/regions to identify important SNPs that are unique to the Arabian Peninsula population. We summed up the numbers of alleles over the populations/regions except for the Arabian Peninsula to compare allele frequencies between the Arabian Peninsula population and the other populations/regions (which will be referred to as "other regions" hereafter). In this study, we ignored the differences among populations in “other regions.”

### Identification of T2D-related SNPs

To obtain T2D-related SNPs, we searched the ClinVar database (https://www.ncbi.nlm.nih.gov/clinvar/) with the keyword ‘”type 2 diabetes”[dis] OR “diabetes mellitus type 2”[dis]’. We extracted SNPs that were common among gnomAD exome, GME Variome, and the result of keyword search at the ClinVar database based on the chromosome, position, reference allele, and alternative allele. ClinVar provides clinical significance, which we used to evaluate the clinical significance of SNPs.

### Statistical analyses

To compare the allele frequencies and genotype frequencies between the Arabian Peninsula and other regions, we divided the data into the Arabian Peninsula and the other regions.

The observed number of each allele in the Arabian Peninsula was calculated as follows:

RC(AP)=ReferenceallelecountintheArabianpeninsulapopulation

AC(AP)=AlternativeallelecountintheArabianpeninsulapopulation

For the other regions, the calculation was as follows:

$$RC(Others)=Reference\;allele\;count\;among\;other\;regions=\sum_{i}Reference\;allele\;count\;of\;population\;i$$

$$AC(Others)=Alternative\;allele\;count\;among\;other\;regions=\sum_{i}Alternative\;allele\;count\;of\;population\;i$$

where i is the population except for the population in the Arabian Peninsula.

Then, the expected allele counts in the Arabian Peninsula were calculated as follows:

$$Expected\;RC(AP)=(RC(AP)+AC(AP))\times \;\frac{RC(Others)}{RC(Others)+AC(Others)}$$

$$Expected\;AC(AP)=(RC(AP)+AC(AP))\times \;\frac{AC(Others)}{RC(Others)+AC(Others)}$$

Additionally, we calculated the expected values for the genotype count using the same method. These expected allele counts represented the allele counts when the allele frequencies of each population were equal.

We performed Fisher’s exact test for each SNP to identify statistically significant SNPs that were not independent for the population, allele counts, or genotype counts. The Benjamini–Hochberg method was used with a q-value of 0.05 for the correction of multiple tests. For SNPs that were statistically significant, we further examined them using a residual analysis to reveal the differences between the observed counts and expected counts.

## Results and discussion

### Integration of gnomAD exome and GME Variome

Before the GME Variome data became available, genotype data for the Middle Eastern populations were scarce. In 2016, an exome project on the Greater Middle Eastern region was finally published [9], which allowed for analyses of worldwide genotype data including Middle Eastern populations by integrating gnomAD exome and GME Variome data.

**Fig 1:**
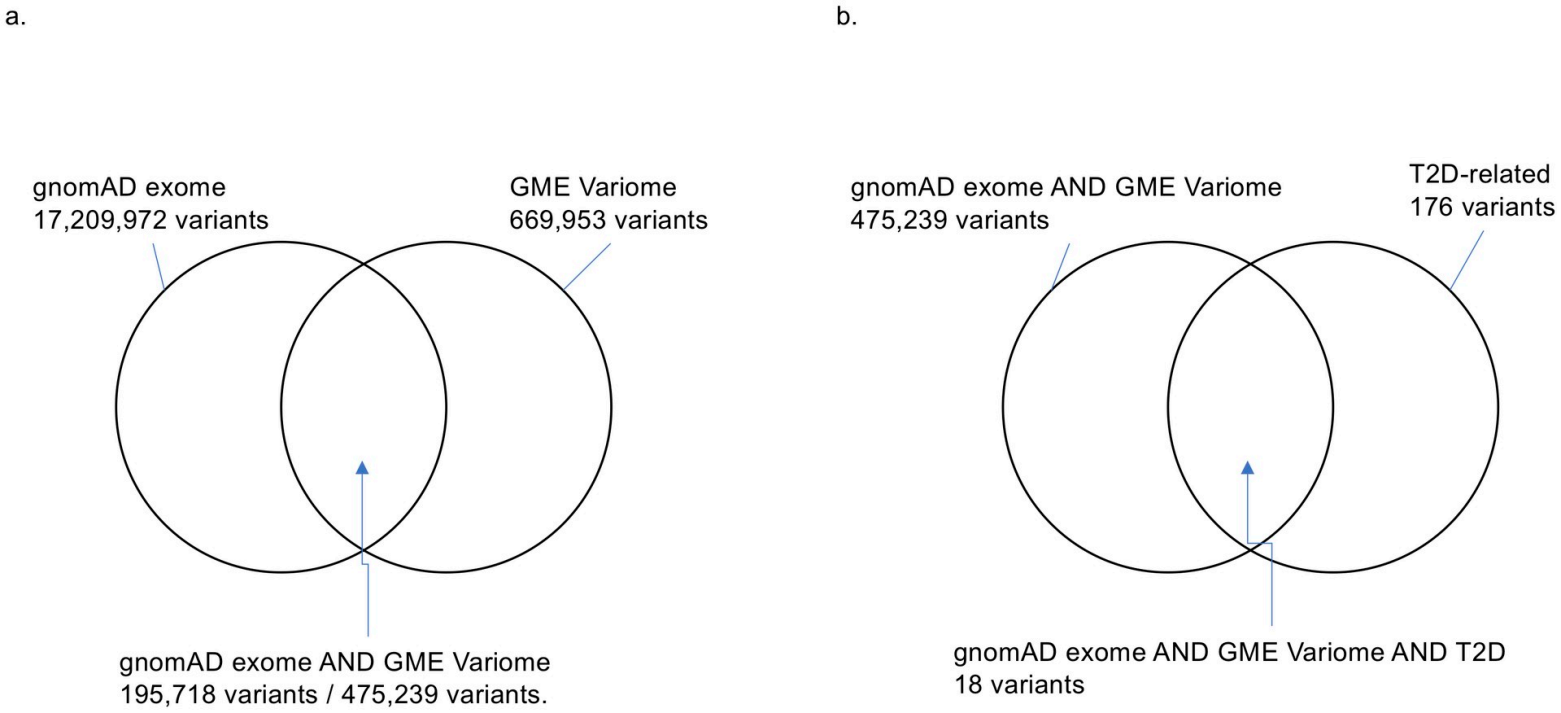
Number of variants. (a) The intersection between the gnomAD exome and GME Variome data. 475,239 variants commonly exist in two databases. After the eliminating rare variants, 195,718 variants remained. (b) Intersection among the gnomAD exome, GME Variome, and T2D-related variants.

We downloaded 17,209,972 and 669,953 variants from gnomAD exome and GME Variome, respectively. We merged the genotype information for gnomAD exome and GME Variome and obtained 475,239 variants for 15 populations/regions. After eliminating the rare variants, we obtained 195,718 variants (Fig 1a). Then, we summed up the numbers of alleles for the Arabian Peninsula and other regions and calculated the expected value for each variant. The allele frequency spectra of overlapped variants showed similar tendencies among the Arabian Peninsula and other regions (S1a Fig) and also among each population/region (S1b Fig).

### Significant T2D-related SNPs in the Arabian Peninsula

**Fig 2:**
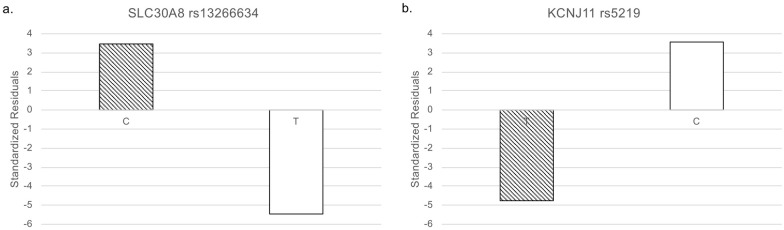
Standardized residuals of alleles. White: non-risk allele and diagonal stripes: risk allele. (a) *SLC30A8* rs13266634. (b) *KCNJ11* rs5219.

In addition to the genotype data, we integrated clinical information via ClinVar to reveal genetic features related to T2D. We downloaded 176 T2D-related variants from ClinVar as of January 17, 2021. Only 18 SNPs were common among the three databases (Table 1 and Fig 1b). To identify SNPs that were specifically biased in the Arabian Peninsula subpopulations, we performed Fisher’s exact test on these 18 SNPs. Even though the variants were mainly derived from populations outside of the Middle East, we found that six SNPs were statistically significant (Table 2). Two of the six SNPs were clinically significant, as described below. Among the two statistically and clinically significant SNPs, residual analysis revealed that a variant, rs13266634, increased the number of the risk allele (Fig 2).

**Table 1:** List of T2D-related SNPs.

rs-number	Chr	Position	REF	ALT	Change	gene	protein	Clinical significance
rs41265094	2	227661003	C	G	c.2452G>C (p.Gly818Arg)	IRS1	insulin receptor substrate 1	Likely benign
rs1801276	2	227661921	C	G	c.1534G>C (p.Ala512Pro)	IRS1	insulin receptor substrate 1	Benign
rs1044498	6	132172368	A	C	c.517A>C (p.Lys173Gln)	ENPP1	ectonucleotide pyrophosphatase/phosphodiesterase 1	Benign
rs1799999	7	113518434	C	A	c.2713G>T (p.Asp905Tyr)	PPP1R3A	protein phosphatase 1 regulatory subunit 3A	Uncertain significance
rs13266634	8	118184783	C	T	c.973C>T (p.Arg325Trp)	SLC30A8	solute carrier family 30 member 8	risk factor
rs5219	11	17409572	T	C	c.67A>G (p.Lys23Glu)	KCNJ11	potassium inwardly rectifying channel subfamily J member 11	drug response
rs566325901	12	121177120	A	G	c.1108A>G (p.Met370Val)	ACADS	acyl-CoA dehydrogenase short chain	Conflicting interpretations of pathogenicity
rs121434581	17	7189048	G	A	c.1147G>A (p.Val383Ile)	SLC2A4	solute carrier family 2 member 4	Uncertain significance
rs1801483	17	79767715	G	A	c.118G>A (p.Gly40Ser)	GCGR	glucagon receptor	Benign
rs41309435	19	40741862	C	A	c.1110G>T (p.Pro370 =)	AKT2	AKT serine/threonine kinase 2	Benign
rs776435289	19	40743996	C	G	c.711G>C p.Leu237 =)	AKT2	AKT serine/threonine kinase 2	Likely benign
rs199761368	19	40744854	G	A	c.666C>T (p.His222 =)	AKT2	AKT serine/threonine kinase 2	Benign
rs139125633	19	40761070	G	A	c.282C>T (p.Asp94 =)	AKT2	AKT serine/threonine kinase 2	Likely benign
rs35588791	19	40762915	G	A	c.93C>T (p.Ser31 =)	AKT2	AKT serine/threonine kinase 2	Benign
rs1799816	19	7125518	C	T	c.3034G>A (p.Val1012Met)	INSR	insulin receptor	Conflicting interpretations of pathogenicity
rs142204928	20	43043159	G	A	c.439G>A (p.Val147Ile)	HNF4A	hepatocyte nuclear factor 4 alpha	Conflicting interpretations of pathogenicity
rs147638455	20	43058267	A	G	c.1321A>G (p.Ile441Val)	HNF4A	hepatocyte nuclear factor 4 alpha	Uncertain significance
rs2076026	20	43942676	T	C	c.759T>C (p.Ala253 =)	RBPJL	recombination signal binding protein for immunoglobulin kappa J region like	Benign

Positions are on the Assembly GRCh37. Clinical significance is based on ClinVar. Chr: Chromosome number, REF: Reference allele, ALT: Alternative allele, Change: Substitutions of nucleotide and amino acid.

**Table 2:** Results of Fisher’s exact test for T2D-related SNPs.

	Allele Count					Genotype Count						
rs-number	Observed		Expected		Fisher	Observed			Expected			Fisher
	REF	ALT	REF	ALT		REF/REF	REF/ALT	ALT/ALT	REF/REF	REF/ALT	ALT/ALT	
rs41265094	342	0	339	3	0.249	171	0	0	168	3	0	0.248
rs1801276	342	0	337	5	0.062	171	0	0	166	5	0	0.061
rs1044498 #	245	97	275	67	0.009	92	61	18	115	45	11	0.037
rs1799999	285	57	267	75	0.099	117	51	3	109	49	13	0.039
rs13266634 *#§	298	44	244	98	0.000	130	38	3	88	68	15	0.000
rs5219 *#§	70	272	123	219	0.000	12	46	113	23	76	72	0.000
rs566325901	341	1	341	1	1.000	170	1	0	170	1	0	1.000
rs121434581	337	5	341	1	0.217	167	3	1	170	1	0	0.371
rs1801483 #§	225	15	238	2	0.002	106	13	1	118	2	0	0.003
rs41309435	342	0	341	1	1.000	171	0	0	170	1	0	1.000
rs776435289	342	0	342	0	1.000	171	0	0	171	0	0	1.000
rs199761368	341	1	341	1	1.000	170	1	0	170	1	0	1.000
rs139125633	342	0	342	0	1.000	171	0	0	171	0	0	1.000
rs35588791	342	0	341	1	1.000	171	0	0	170	1	0	1.000
rs1799816	335	7	339	3	0.340	164	7	0	168	3	0	0.337
rs142204928	341	1	341	1	1.000	170	1	0	170	1	0	1.000
rs147638455 #§	331	11	342	0	0.001	160	11	0	171	0	0	0.001
rs2076026 #	329	13	311	31	0.007	159	11	1	143	25	3	0.016

**Fig 3:**
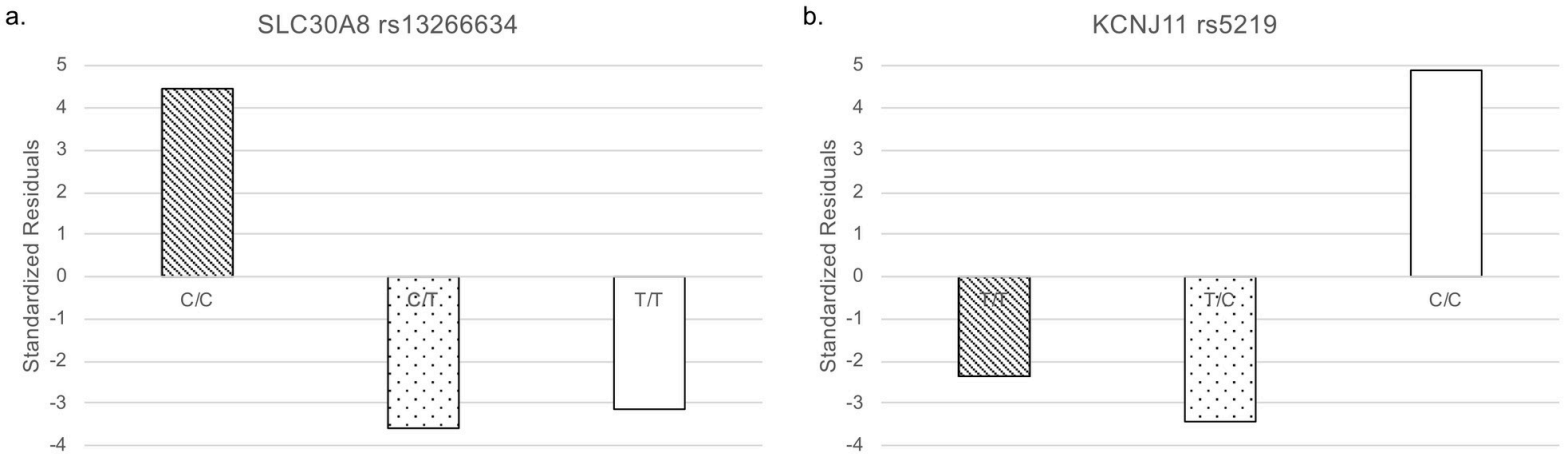
Standardized residuals of genotypes. White: homozygote non-risk alleles, dotted: heterozygote, and diagonal stripes: homozygote of risk alleles. (a) *SLC30A8* rs13266634. (b) *KCNJ11* rs5219.

Interestingly, for rs13266634, the homozygous risk genotype increased whereas the heterozygous genotype decreased in the Arabian Peninsula (Fig 3). The remaining SNP was rs5219. The number of this risk allele decreased in the Arabian Peninsula (Fig 2).

The numbers of alleles and numbers of genotypes were tested. In the rs-number column, * represents the clinically significant SNP, # represents statistically significant SNP according to Fisher’s exact test the allele count, and § represents statistically significant SNP by Fisher’s exact test using the genotype count. The Benjamini–Hochberg method was used with a q-value of 0.05 for the correction of multiple tests. Observed and expected numbers of alleles and genotypes are shown. REF: reference allele, ALT: alternative allele, REF/REF: homozygote of reference allele, REF/ALT: heterozygote, ALT/ALT: homozygote of alternative allele, Fisher: p-value of Fisher’s exact test.

#### *SLC30A8* rs13266634

rs13266634 is located on chromosome 8 (8q24.11) in the *SLC30A8* gene, which encodes solute carrier family 30 member 8. It is also known as Zinc Transporter 8 (ZnT8). Zinc plays an important role in insulin processing and storage. ZnT8 is specifically expressed in the pancreas and transports Zn^2+^ from the cytosol to the insulin-secreting granules. A nonsynonymous variant, rs13266634, is enriched in diabetic patients. This SNP changes a tryptophan residue into to an arginine residue (R325W). R325 carriers have an increased risk of developing T2D [20].

The frequency of the risk allele of rs13266634 is higher in the Arabian Peninsula than in other regions. The frequency of the homozygote of this risk allele has increased in the Arabian Peninsula compared with that in the other regions. However, the frequency of the heterozygote has decreased in the Arabian Peninsula compared with that in the other regions. Our results suggest that this SNP is fixed to the risk allele for T2D among individuals residing in the Arabian Peninsula.

#### *KCNJ11* rs5219

rs5219 is located on chromosome 11 (11p15.1) in the *KCNJ11* gene. The *KCNJ11* and *ABCC8* genes are next to each other and encode potassium voltage-gated channel subfamily J member 11 and ATP binding cassette subfamily C member 8. The KCNJ11 and ABCC8 proteins in the ATP-sensitive potassium (KATP) channel mediate insulin secretion. The rs5219 may alter the charge of the ATP binding region and decrease channel sensitivity [21]. The rs5219 polymorphism is a risk factor for developing T2D in Caucasians and in some Asian populations [21]. East Asian populations were more prone to this disease, as the risk allele (A) was more common in most East Asian patients than in the controls [21].

The frequency of the risk allele of rs5219 in the Arabian Peninsula is lower than that in other regions. Our data revealed that the risk for this SNP is low among individuals from the Arabian Peninsula.

## Conclusion

In this study, we found that two clinically significant T2D-related SNPs were statistically significant in the Arabian Peninsula. These SNPs are located in genes that are associated with metal uptake. Imbalance levels of essential trace metals affect T2D [22], and our results infer that metals may be one of the factors for the development of T2D for individuals in the Arabian Peninsula. It is well-known that dates, a popular fruit in the Arabian Peninsula, contain plenty of minerals. Our results show that there were deviations in the allele frequencies among the Arabian Peninsula and other regions. The integrated dataset is useful to find such genetic deviations among populations. Our analyses contribute to and provide guidance for clinical research of type 2 diabetes in the Arabian Peninsula populations. Our approach could elucidate the genetic features of other populations and diseases with known SNPs and novel SNPs from future studies such as GWAS, which will be helpful for future studies and clinical applications.

## Supporting information

S1 Fig Allele frequency spectrum. x-axis shows allele frequency, and y-axis shows the number of variants. (a) Allele Frequency Spectrum of the Arabian Peninsula and other regions. (b) Allele Frequency Spectrum of each populations/regions.
(PDF)

## References

[1] International Diabetes Federation. Type 2 diabetes 2020. https://www.idf.org/aboutdiabetes/type-2-diabetes.html.

[2] Dean L, McEntyre J. The Genetic Landscape of Diabetes 2021. https://www.ncbi.nlm.nih.gov/books/NBK1667/.

[3] Mokdad AH, Jaber S, Aziz MIA, AlBuhairan F, AlGhaithi A, AlHamad NM, et al. The state of health in the Arab world, 1990–2010: an analysis of the burden of diseases, injuries, and risk factors. The Lancet. 2014;383(9914):309–20. 10.1016/s0140-6736(13)62189-3 24452042

[4] International Diabetes Federation. IDF DIABETES ATLAS Ninth edition 2019. 2019. 35914061

[5] International HapMap Consortium. A second generation human haplotype map of over 3.1 million SNPs. Nature. 2007;449(7164):851–61. Epub 2007/10/19. 10.1038/nature06258 . 17943122 PMC2689609

[6] Cavalli-Sforza LL. The Human Genome Diversity Project: past, present and future. Nature Review Genetics. 2005;6:333–40. 10.1038/nrg1596 15803201

[7] The 1000 Genomes Project Consortium. A global reference for human genetic variation. Nature. 2015;526(7571):68–74. 10.1038/nature15393 26432245 PMC4750478

[8] Karczewski KJ, Francioli LC, Tiao G, Cummings BB, Alfoldi J, Wang Q, et al. The mutational constraint spectrum quantified from variation in 141,456 humans. Nature. 2020;581(7809):434–43. Epub 2020/05/29. 10.1038/s41586-020-2308-7 . 32461654 PMC7334197

[9] Scott EM, Halees A, Itan Y, Spencer EG, He Y, Azab MA, et al. Characterization of Greater Middle Eastern genetic variation for enhanced disease gene discovery. Nature Genetics. 2016;48(9):1071–6. 10.1038/ng.3592 27428751 PMC5019950

[10] Buniello A, MacArthur JAL, Cerezo M, Harris LW, Hayhurst J, Malangone C, et al. The NHGRI-EBI GWAS Catalog of published genome-wide association studies, targeted arrays and summary statistics 2019. Nucleic Acids Res. 2019;47(D1):D1005–D12. Epub 2018/11/18. 10.1093/nar/gky1120 . 30445434 PMC6323933

[11] Scott RA, Scott LJ, Magi R, Marullo L, Gaulton KJ, Kaakinen M, et al. An Expanded Genome-Wide Association Study of Type 2 Diabetes in Europeans. Diabetes. 2017;66(11):2888–902. Epub 2017/06/02. 10.2337/db16-1253 . 28566273 PMC5652602

[12] Xue A, Wu Y, Zhu Z, Zhang F, Kemper KE, Zheng Z, et al. Genome-wide association analyses identify 143 risk variants and putative regulatory mechanisms for type 2 diabetes. Nat Commun. 2018;9(1):2941. Epub 2018/07/29. 10.1038/s41467-018-04951-w . 30054458 PMC6063971

[13] Shu XO, Long J, Cai Q, Qi L, Xiang YB, Cho YS, et al. Identification of new genetic risk variants for type 2 diabetes. PLoS Genet. 2010;6(9):e1001127. Epub 2010/09/24. 10.1371/journal.pgen.1001127 . 20862305 PMC2940731

[14] Spracklen CN, Horikoshi M, Kim YJ, Lin K, Bragg F, Moon S, et al. Identification of type 2 diabetes loci in 433,540 East Asian individuals. Nature. 2020;582(7811):240–5. Epub 2020/06/06. 10.1038/s41586-020-2263-3 . 32499647 PMC7292783

[15] Cook JP, Morris AP. Multi-ethnic genome-wide association study identifies novel locus for type 2 diabetes susceptibility. Eur J Hum Genet. 2016;24(8):1175–80. Epub 2016/05/18. 10.1038/ejhg.2016.17 . 27189021 PMC4947384

[16] Myles S, Davison D, Barrett J, Stoneking M, Timpson N. Worldwide population differentiation at disease-associated SNPs. BMC Medical Genomics. 2008;1(1). 10.1186/1755-8794-1-22 18533027 PMC2440747

[17] Popejoy AB, Fullerton SM. Genomics is failing on diversity. Nature. 2016;538:161–4. 10.1038/538161a 27734877 PMC5089703

[18] B CD., Burchard EG, De La Vega FM. Genomics for the world. Nature. 2011;475:163–5. 10.1038/475163a 21753830 PMC3708540

[19] Need AC, Goldstein DB. Next generation disparities in human genomics: concerns and remedies. Trends Genet. 2009;25(11):489–94. Epub 2009/10/20. 10.1016/j.tig.2009.09.012 . 19836853

[20] Chabosseau P, Rutter GA. Zinc and diabetes. Archives of Biochemistry and Biophysics. 2016;611:79–85. 10.1016/j.abb.2016.05.022 27262257

[21] Haghvirdizadeh P, Mohamed Z, Abdullah NA, Haghvirdizadeh P, Haerian MS, Haerian BS. KCNJ11: Genetic Polymorphisms and Risk of Diabetes Mellitus. Journal of Diabetes Research. 2015;2015:1–9. 10.1155/2015/908152 26448950 PMC4584059

[22] Khan AR, Awan FR. Metals in the pathogenesis of type 2 diabetes. Journal of Diabetes & Metabolic Disorders. 2014;13:16. 10.1186/2251-6581-13-16 24401367 PMC3916582

