# Supplementary figures and images for: Significant variants of type 2 diabetes in the Arabian Region through an Integration of exome databases
Source: PLoS One. 2021 Apr 13;16(4):e0249226. doi: 10.1371/journal.pone.0249226 (PMC8043376; doi:10.1371/journal.pone.0249226)

a.

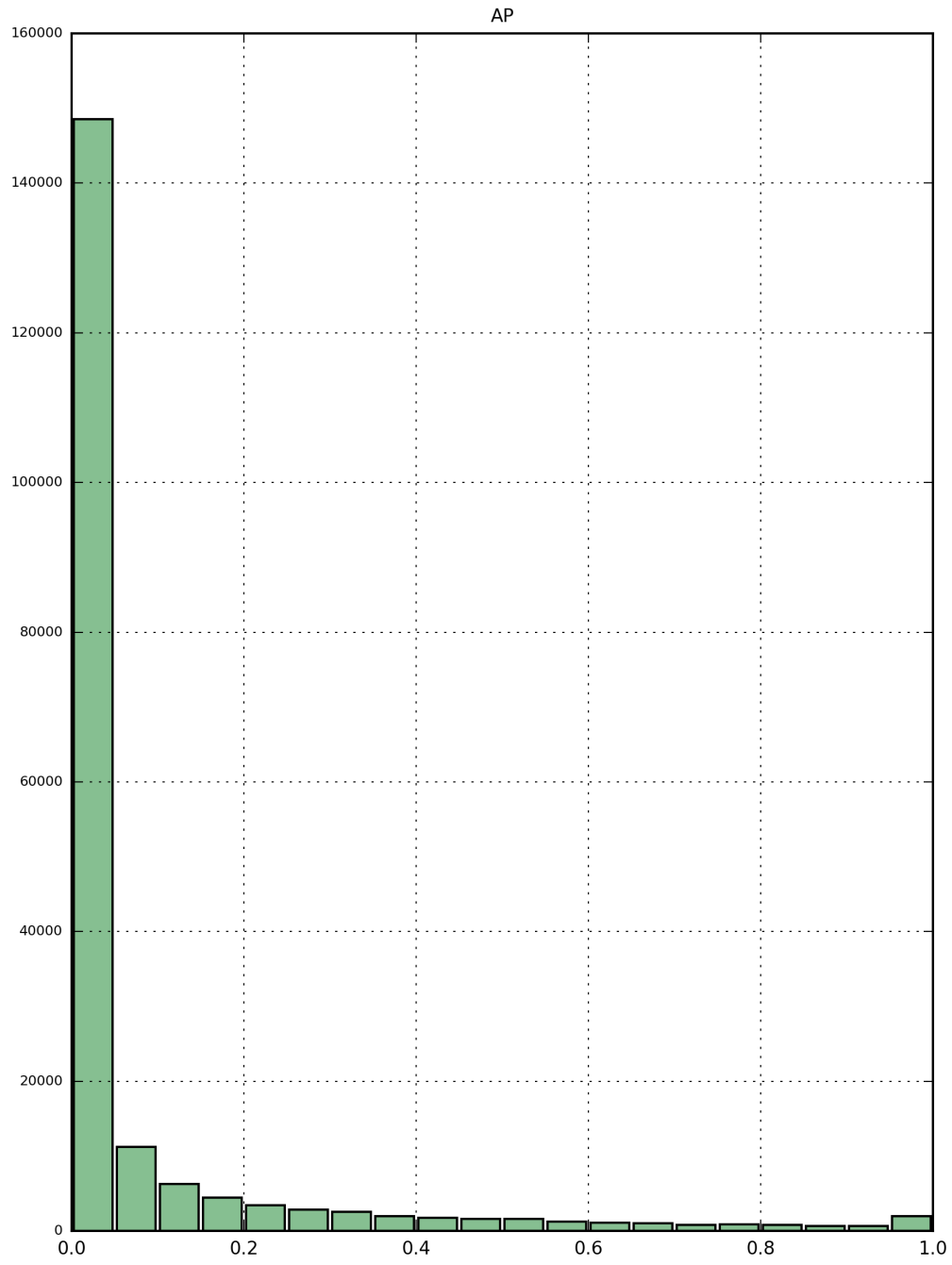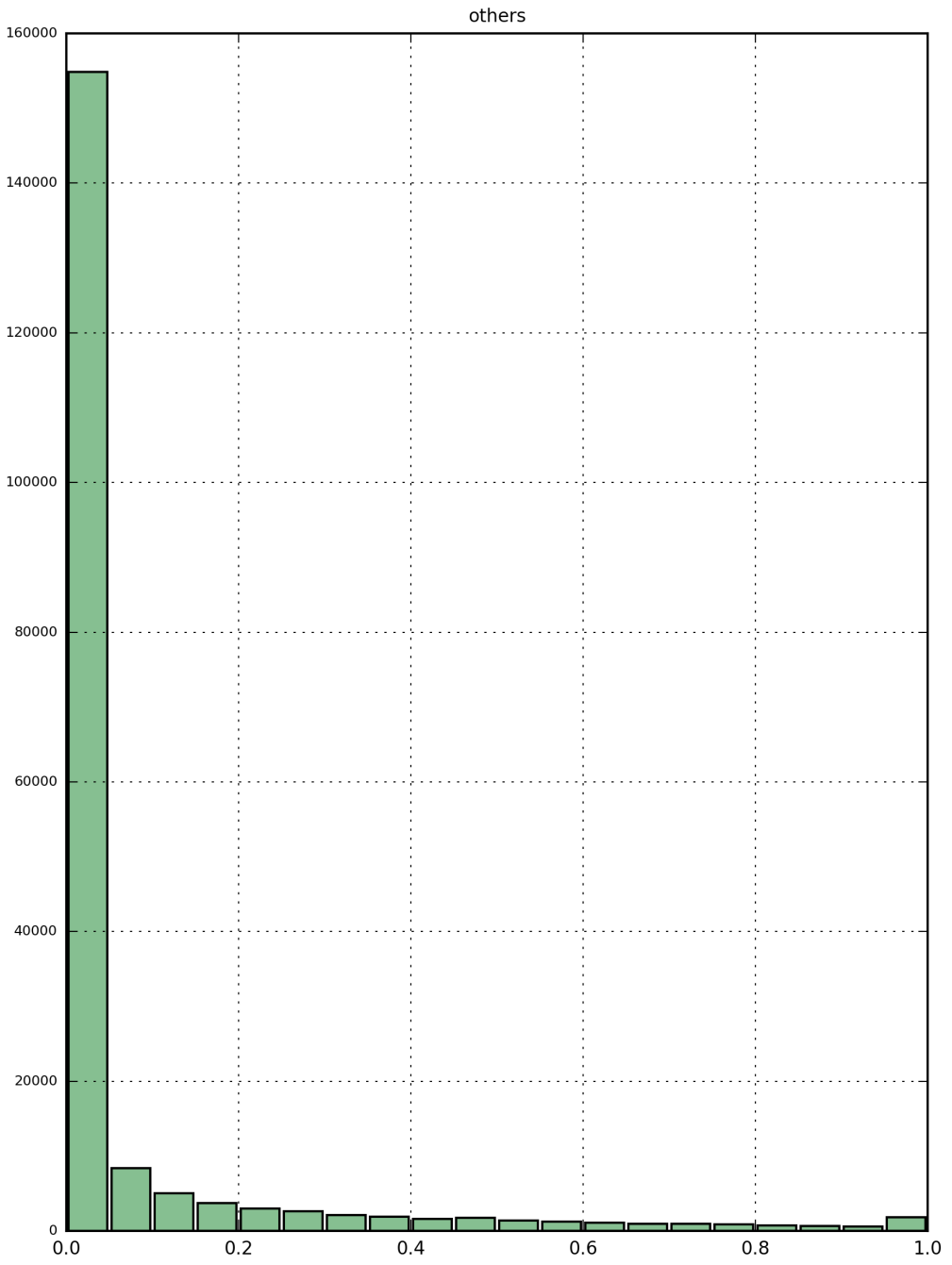

b.

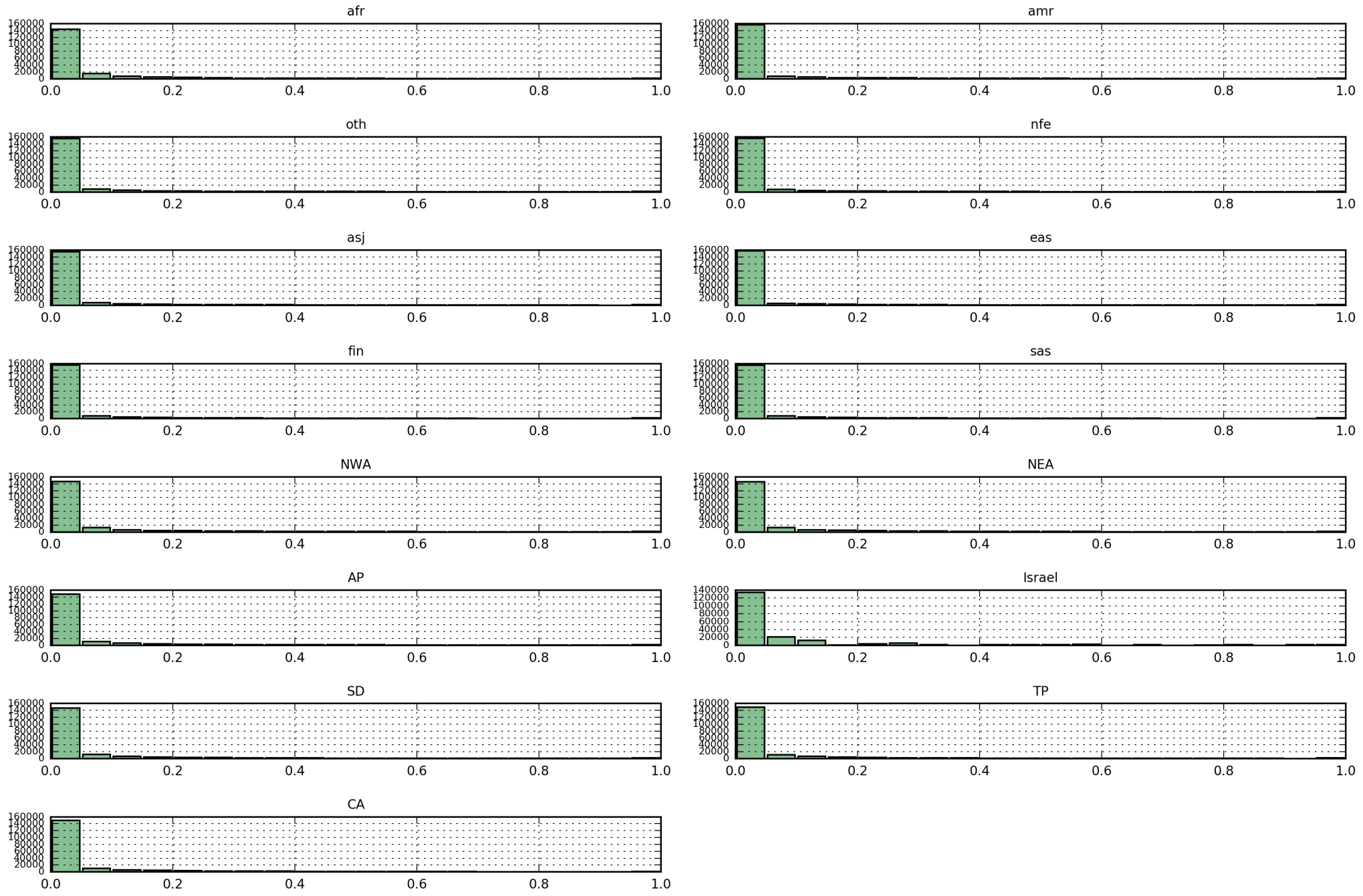

Supplement: S1 Fig — x-axis shows allele frequency, and y-axis shows the number of variants. (a) Allele Frequency Spectrum of the Arabian Peninsula and other regions. (b) Allele Frequency Spectrum of each populations/regions. (PDF) [file pone.0249226.s001.pdf]
